# Response to letter regarding variants in the interferon regulatory factor 5 gene confer genetic risk for systemic lupus erythematosus in a Han Chinese population

**DOI:** 10.1080/07853890.2026.2643040

**Published:** 2026-03-17

**Authors:** Wenqi Xu, Rongzeng Liu, Xiaofei Shi

**Affiliations:** a Department of Immunology, College of Basic Medicine and Forensic Medicine, Henan University of Science and Technology, Luoyang, China; b Department of Rheumatology and Immunology, The First Affiliated Hospital, and College of Clinical Medicine of Henan University of Science and Technology, Luoyang, China; c Key Laboratory of Clinical Immunology and Inflammatory Diseases, Luoyang, China

We acknowledge the thoughtful comments provided by Dr. Panda and Dr. Sharma [1] on our recent article [2] and welcome the opportunity to clarify several methodological aspects of our study. This scholarly dialogue offers valuable perspectives for advancing rigor in genetic association research.

With regard to Hardy-Weinberg equilibrium (HWE), the correspondents note that the HWE *p*-values for rs10954213 (*p* = 0.093) and rs2004640 (*p* = 0.081) in our control group approach the conventional significance threshold of 0.05 and suggest cautious interpretation. In genetic association studies, a *p*-value greater than 0.05 is conventionally considered consistent with HWE. Both values exceed this threshold and therefore do not indicate statistically significant deviation. While the *p*-values are relatively modest, they are most plausibly attributable to sampling fluctuation in a moderately sized cohort (*n* = 246) rather than systematic genotyping error or population stratification. All participants were recruited from a single geographic region in Northern China and were of Han Chinese ethnicity, minimizing potential stratification bias. Genotyping call rates exceeded 98% for both variants, and no excess or deficiency of heterozygotes was observed. Furthermore, the minor allele frequencies were consistent with publicly available East Asian reference datasets. Collectively, these findings support the genetic representativeness and reliability of the control cohort.

Regarding the genotyping methodology, the Letter raises concerns about the number of PCR amplification cycles and the validation approach. Upon re-examining our laboratory documentation, we confirm that the final genotyping experiments were performed using 28 amplification cycles. The 34-cycle condition referenced in the manuscript corresponded to an optimization stage and was not applied in the formal genotyping dataset used for analysis. All association results reported in the study were derived from the validated 28-cycle protocol.

The 28-cycle protocol was optimized to ensure specific amplification without nonspecific products. To further ensure genotyping reliability, duplicate genotyping was performed on more than 20% of randomly selected samples, yielding 100% concordance. All electrophoretic results were independently reviewed by two researchers to minimize interpretation bias. PCR products were resolved using 2% agarose gel electrophoresis, providing clear discrimination of target bands, and strict negative controls were included in each run to monitor for contamination. Only high-quality DNA samples (A260/A280 ratios between 1.8 and 2.0) were included in the analysis. While sequencing-based confirmation represents an alternative validation strategy, the combination of extensive duplicate testing, independent scoring, and optimized assay conditions provides strong support for the accuracy and reproducibility of the genotyping data presented.

The correspondents also note an apparent inconsistency between the Bonferroni-adjusted *p*-values presented in Table 4 and the statement in the Methods section describing the use of the Benjamini-Hochberg false discovery rate (FDR) approach. We clarify that two complementary correction strategies were applied in different analytical contexts. For the primary genotype-disease association analyses, Bonferroni correction was used to conservatively adjust for the multiple genetic models tested per SNP. For exploratory genotype-phenotype analyses involving a larger number of correlated comparisons, the Benjamini-Hochberg FDR method was employed to control for false discoveries while maintaining reasonable statistical power. We acknowledge that clearer differentiation of these strategies in the Methods section would have enhanced clarity. Importantly, the principal associations for rs2004640 and rs10954213 remained statistically significant after conservative Bonferroni adjustment, indicating that the primary conclusions are not dependent on less stringent statistical thresholds.

Taken together, the conformity to HWE, high genotyping reproducibility, conservative correction of primary analyses, and consistency with established functional evidence for IRF5 variants collectively support the robustness of our principal findings. We appreciate the opportunity to clarify these points and thank the correspondents for highlighting important considerations that contribute to methodological transparency in genetic association research.

## Data Availability

No new data were generated during this study.
